# *Leishmania mexicana* pathogenicity requires flagellar assembly but not motility

**DOI:** 10.1080/21505594.2025.2521478

**Published:** 2025-07-02

**Authors:** Tom Beneke, Rachel Neish, Carolina M. C. Catta-Preta, James Smith, Jessica Valli, Ciaran J. McCoy, Andreia Albuquerque-Wendt, Jeremy C. Mottram, Eva Gluenz

**Affiliations:** a Sir William Dunn School of Pathology, University of Oxford, Oxford, UK; b Department of Cell and Developmental Biology, Biocentre, University of Würzburg, Würzburg, Germany; c York Biomedical Research Institute, Department of Biology, University of York, York, UK; d Laboratory of Parasitic Diseases, National Institute of Allergy and Infectious Diseases, National Institutes of Health, Bethesda, MD, USA; e School of Infection and Immunity, University of Glasgow, Glasgow, UK; f Harry Perkins Institute of Medical Research, Perth, Australia; g Edinburgh Super Resolution Imaging Consortium, Institute of Biological Chemistry, Biophysics and Bioengineering, School of Engineering and Physical Sciences, Heriot-Watt University, Edinburgh, UK; h Department of Biomedical Sciences, Institute of Tropical Medicine, Antwerp, Belgium; i Institute of Cell Biology, University of Bern, Bern, Switzerland; j Global Health and Tropical Medicine, Instituto de Higiene e Medicina Tropical, Universidade Nova de Lisboa, Lisbon, Portugal; k Department of Parasitology, Faculty of Science Charles University, Prague, Czech Republic; l Parasite Chemotherapy Unit, Swiss Tropical and Public Health Institute, Allschwil, Switzerland

**Keywords:** *Leishmania*, virulence, motility, flagella, CRISPR screen

## Abstract

Protists of the order Trypanosomatida possess a single multifunctional flagellum, which powers cellular displacement and mediates attachment to tissues of the arthropod vector. The kinetoplastid flagellar cytoskeleton consists of a nine-microtubule doublet axoneme; further structural elaborations, which can vary between species and life cycle stages, include the assembly of axonemal dynein complexes, a pair of singlet microtubules and the extra-axonemal paraflagellar rod. The intracellular amastigote forms of *Leishmania* spp. build a short, non-motile cilium whose function has remained enigmatic. Here, we used a panel of 25 barcoded promastigote cell lines, including mutants lacking genes encoding flagellar assembly proteins, axonemal proteins required for normal motility, or flagellar membrane proteins to examine how these defects impact on their virulence in macrophages and mice. Mutants lacking the intraflagellar transport (IFT) protein 88 were avirulent indicating that assembly of a flagellum is necessary to allow for *Leishmania* survival in a mammalian host. A similarly severe loss of virulence was observed upon deletion of *BBS2*, a core component of the BBSome complex, which may act as a cargo adapter for IFT. By contrast, promastigotes that were unable to beat their flagella due to loss of core axonemal proteins could establish and sustain an infection and only showed a small reduction of parasite burden *in vivo* compared to the parental cell lines. These results confirm that flagellar motility is not necessary for mammalian infection, but flagellum assembly and the integrity of the BBSome are essential for pathogenicity.

## Introduction

Protozoan parasites of the order Trypanosomatida remain a threat to human health in vast regions of the globe, with an estimated one billion people at risk of infection. Incomplete knowledge of pathogenicity mechanisms and host–pathogen interactions is still considered an impediment to better treatment [1]. Successful completion of their life cycles depends on their ability to establish infections within the blood-feeding arthropod vectors that transmit them, surviving and proliferating in their specific niches within the vertebrate hosts and eventually re-entering a suitable vector. During the course of those life cycles, the flagellum of the kinetoplastid cell serves multiple functions including motility, attachment and sensing (reviewed in [2]). Their motile flagellum has a canonical 9 + 2 microtubule axoneme adjoined by a paraflagellar rod (PFR) specific to Euglenozoan taxa. Eukaryotic flagella (also called cilia) are built from a basal body, docked at the base of the flagellar pocket (FP), by a bi-directional intraflagellar transport (IFT) system that delivers proteins to the tip of the growing flagellum for the growth and maintenance of the structure [3,4]. In the following, the term flagellar assembly refers to the IFT-dependent process that generates the functional flagellum of wild-type *Leishmania*. The BBSome, a subcomplex of the IFT system [5] acts as a cargo adaptor specifically required for controlling the transport of select membrane proteins into and out of cilia [6]. Molecular components required for flagellar assembly, structure and beat generation are highly conserved across ciliated eukaryotes [7–12]. This broad conservation and advanced understanding of many of the key mechanisms that underpin ciliogenesis and organelle function allows for targeted engineering of parasite cell lines with specific deficiencies in flagellum assembly (by blocking IFT) or movement (by removal of cytoskeletal proteins that generate or modulate the flagellar beat), to discover how the flagellum contributes to the parasites’ fitness in the insect vector and to test its contributions to pathogenicity.

Genetic studies on *Trypanosoma brucei* have revealed complex dependencies between cell morphogenesis and flagellar assembly during the cell cycle [13–15] and established a vital role for motility in sustaining an infection in the mammal and for completion of its life cycle in the insect vector. *T. brucei* bloodstream forms are particularly sensitive to perturbations of flagellar motility: downregulation or mutation of proteins required for normal flagellar beating leads to rapid and catastrophic failures of cell division and cell death *in vitro* [8,16], and clearance from infected mice *in vivo* [17,18]. In tsetse flies, deletion mutants for the axonemal dynein intermediatechain DNAI1 that lost their ability to swim forward [19] were unable to migrate from the gut to the salivary glands [20].

*Leishmania* promastigotes in the sand fly have a long motile flagellum, which shares fundamental mechanisms with the motile *T. brucei* flagellum. One important difference is the fact that the promastigote flagellum is not attached to the *Leishmania* cell body beyond the exit point from the flagellar pocket, which alleviates the *Leishmania* cell from the absolute dependency of cell division on flagellum growth that exists in *T. brucei* [21]. Large-scale phenotyping of *Leishmania* flagellar mutants showed that assembly of a functional motile flagellum was vital for passage through sand flies [12]. Deletion of the central pair-associated protein PF16 produced morphologically normal-looking promastigotes, which were paralysed [22] and incapable of reaching the thoracic region of the sandfly midgut [12]. Removal of the intraflagellar transport (IFT) protein IFT88 or IFT140 [12,21] produced viable promastigotes with no external flagellum, capable of productive cell division *in vitro* but unable to persist in sand flies [12].

The importance of the promastigote flagellum in the infection of mammalian host cells is less clear. Unlike, e.g. apicomplexan parasites, where the actin-dependent motility of the parasite drives host cell invasion [23,24], *Leishmania* are thought to rely fully on the host cell’s own mechanisms of phagocytosis to gain entry [25,26]. Studies on polarized engulfment of the promastigotes and the importance of the parasite flagellum yielded mixed findings: While some studies suggested the flagellum may be involved in establishing contact between the parasite and the host cell [27,28], others found that *Leishmania* enter their host cells with their posterior ends first [29], or with both ends [25,30,31]. Metacyclic promastigotes, which are pre-adapted to survival in the mammalian host, were seen to exhibit a characteristic “run and tumble” movement, which switched to a faster straighter mode of swimming when presented with human macrophages *in vitro*. This led to the hypothesis that chemotactic sensing promotes active movement of the parasite toward its host cell [32]. Moreover, flagellar beating inside the host cell was reported to trigger host cell response pathways that benefited parasite survival [28]. However, flagellar beating was shown not to be required for phagocytosis of *Leishmania* promastigotes *in vitro* [31,33–35] and freshly deposited *Leishmania* promastigotes observed by intra-vital 2-photon microscopy were found to be quite immotile in the skin [36]. To what extent the motile promastigote flagellum contributes to the establishment of successful infections has not been answered conclusively. Nor is it certain what function is served by the radical remodeling the flagellum that occurs when metacyclic promastigotes differentiate to amastigotes: within the first 24 hours of experiencing the differentiation signal, the long motile flagellum is remodeled to a short nonmotile 9 v type axoneme [37]. Its structural similarity with primary cilia suggested that it may act as a sensory organelle [38,39]. Additional functions in organizing amastigote cell morphology, especially the flagellar pocket, which is important for endo- and exocytosis, are also conceivable [40].

Here, we used 15 *L. mexicana* gene deletion mutants with different flagellar defects to test whether possession of a flagellum was essential for infection of mice and whether that flagellum needed to be motile. The effect of perturbing membrane protein trafficking was tested indirectly, through disruption of the BBSome. The combined results from pooled infection assays, and separate infections with three individual mutants, showed that the *IFT88* deletion mutants, which lacked external flagella, were completely avirulent. Mutants for the BBSome subunit BBS2 were also avirulent despite possessing a motile flagellum. By contrast, immotile mutants lacking axoneme proteins were capable of establishing infections and persisted in the mice for at least 6 weeks, albeit with lower parasite burdens measured for Δ*PF16* at the 8-week end point. These results demonstrate that flagellar motility is not essential for the pathogenicity of *Leishmania*.

## Results

### A pooled screen of flagellar mutants indicates that flagellar assembly is important for infection of a mammal but flagellar beating is dispensable

A panel of barcoded gene deletion mutants previously used to test the fitness of mutant promastigotes both in culture and in sand flies [12] was used to assess which type of flagellar defect has an effect on fitness *in vivo*, in a mammalian host.

**Figure 1.**
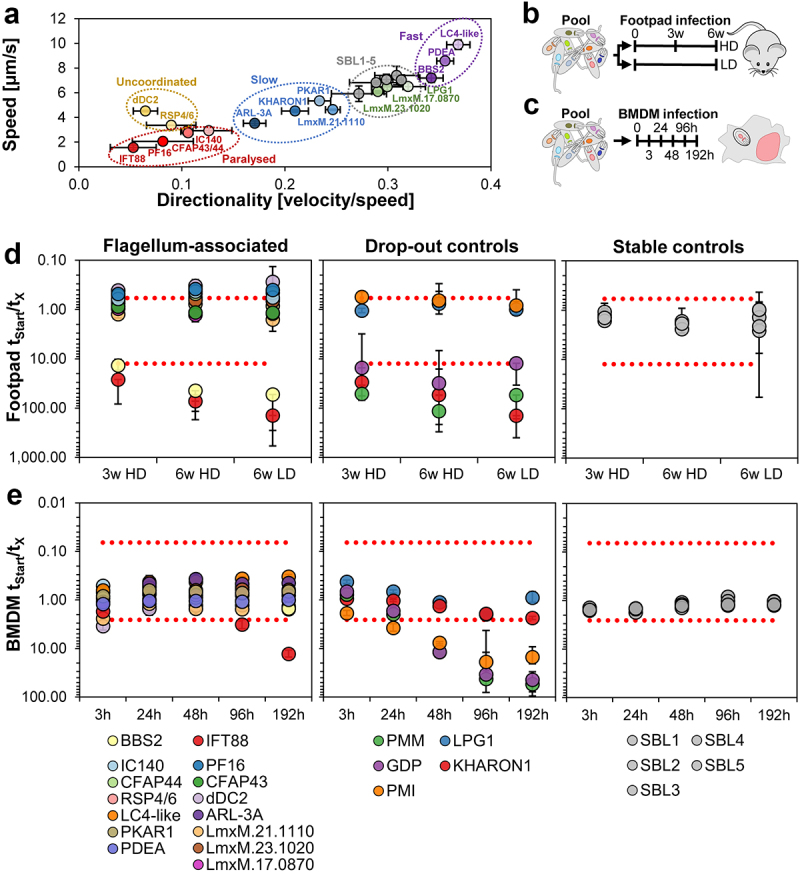
Pooled screen of *L. mexicana* mutants. (a) Average speed [µm/s] plotted against directionality [velocity/speed] for the flagellar mutants and the parental controls (SBL1-5, *L. mex* Cas9 T7 marked with unique 17-nt barcodes in the 18S SSU rRNA locus) included in the bar-seq screens in BMDMs and mice. The motility data is taken from [12]. The motility of mutants ΔGDP, ΔPMI and ΔPMM, which were included in all bar-seq screens as controls, was not measured. (b) Schematic of screen design and timeline. At time point 0, pooled stationary phase promastigotes were used to infect mice either with a high parasite dose [HD] or low dose [LD]. (c) BMDM infections were started from pools as in (b). Time points of genomic DNA isolation are indicated. (d) Bar-seq screen in mice, Y-axis: Change in barcode proportions calculated by dividing the normalized read counts at the start of the assay by the normalized read counts at each DNA isolation time point. X-axis: DNA isolation time points. Each value represents the average from six infected mice. Error bars show standard deviations between replicates. Red dotted lines indicate two standard deviations above and below the average of “stable control” cell lines. Mutants included in the bar-seq screen have been grouped as follows: mutants with flagellum-associated defects, mutants previously reported to show attenuated virulence (“drop-out controls”) and barcoded parental control cell lines (SBL1-5; “stable controls”). (e) Bar-seq screen in BMDMs. Axes and mutant groupings as for (d). Each value represents the average from 3 BMDM samples.

This pool of five barcoded parental cell lines, five control knockout mutants and 15 flagellar mutants with a range of motility defects (Figure 1a, Supplementary Figure S1a-d, Supplementary Table S1) was used to infect murine bone-marrow-derived macrophages (BMDMs) and BALB/c mice.

The persistence of null mutants in the pooled infections was tracked over 8 d in BMDMs and up to 6 weeks in mice. This was done by sequencing the barcoded amplicons prepared from DNA samples of mouse footpad lesions (Figure 1b) and infected BMDMs (Figure 1c). The barcode proportions of the parental cell lines and most mutants remained constant over these observation periods, indicating that most mutants were as fit as the parental controls in the mice and macrophages (Figure 1d,e, Supplementary Table S2). This included all mutants with severe motility defects (the feebly twitching Δ*CFAP43* and Δ*CFAP44*, the uncoordinated Δ*RSP4/6*) and cells with curled up paralysed flagella showing only occasional low-frequency actuation of parts of the flagellum (Δ*IC140* and Δ*PF16* [12,22]). In mice, a strong drop-out phenotype was seen for three knockouts of flagellum-associated proteins (Figure 1d, Supplementary Table S2): Δ*Kharon1*, which lacks a protein required for trafficking of the glucose transporter GT1 to the flagellar membrane, was included in the control group as it had previously been shown to be attenuated *in vivo* [41]. A similar decline was seen for Δ*IFT88* and for Δ*BBS2*. As expected, the control mutants defective in the pathway leading to mannose activation Δ*PMM*, and Δ*GDP-MP* were also attenuated [42] but the barcode proportions for Δ*PMI and* Δ*LPG1* did not diminish over time, indicating that these mutants had parental-like fitness in this pooled infection assay (Figure 1d, Supplementary Table S2). The same results were observed in mice infected with a higher parasite dose (2 × 10^6^ parasites) or a lower dose (2 × 10^5^ parasites). In BMDMs, the control mutants defective in the mannose activation pathway decreased progressively with every time point (Figure 1e). A decline in the proportion of the Δ*IFT88* mutant was noticeable at the final time point in BMDMs. The proportion of Δ*BBS2* mutants remained similar to the parental controls after 8 d in BMDMs (Figure 1e). The longer-term fate of the *Leishmania* in BMDMs could not be assessed, a limitation of the *in vitro* system, which highlights the importance of *in vivo* studies to understand pathogenicity mechanisms.

### Paralysed PF16 mutants are capable of sustaining an infection in vivo

The result of the pooled screen suggested that a motile flagellum is not required for establishing a successful infection in a mouse. However, if flagellar motility aids the initial infection, e.g. by triggering host cell lysosome exocytosis and membrane repair [28], motility-deficient mutants could benefit from the normal flagellar activity of other parasites in the mixed pool that would trigger these host responses. To test whether a motility-defective mutant was able to establish an infection on its own, and persist in a mammal, the Δ*PF16* mutant was used to infect a new set of mice. This mutant was chosen because they exhibited the most severe loss of motility in cells that remained morphologically normal with a long flagellum; we have previously characterized its ultrastructural defect in the axoneme and phenotype in detail [22]. As a control, a copy of *PF16* was re-introduced into the knockout cell line to generate the *PF16*-addback line with restored motility (*PF16*-AB; Supplementary Figure S1e-g). Similarly, addback cell lines were generated for *BBS2* and *IFT88*, to test whether these would rescue the phenotypes of the respective knockout mutants (Supplementary Figure S1e-g). The Cas9 T7 parental cell line and each knockout and addback cell line were used to infect the right footpad of five individual BALB/c mice. The infections were followed weekly by footpad measurements, and parasite burden was calculated for the footpad lesion and lymph nodes, of each mouse.

**Figure 2.**
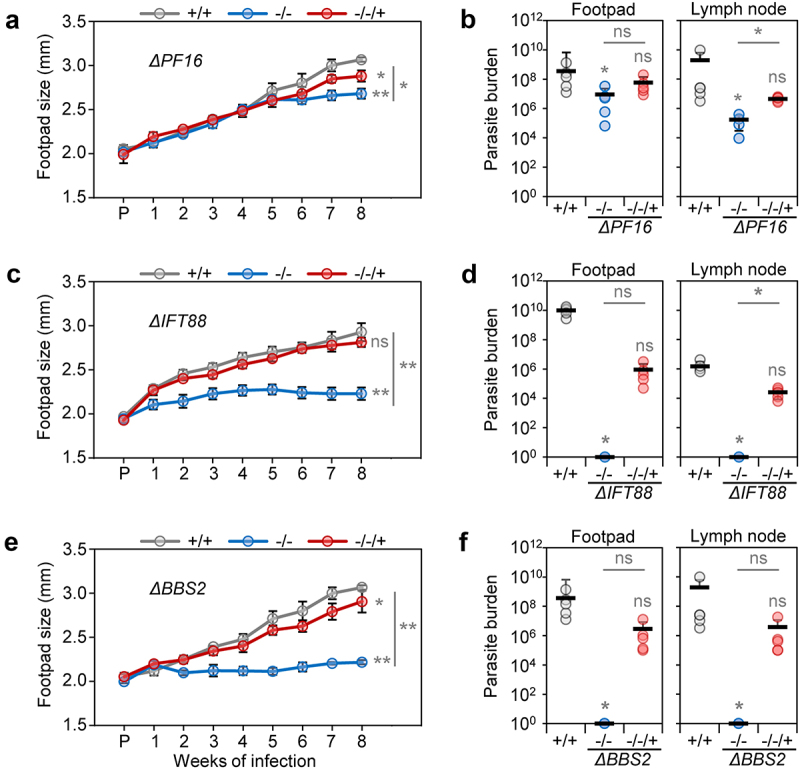
Mouse infections with Δ*PF16*, Δ*IFT88* and Δ*BBS2*. Lesion progression and parasite burden were measured for Cas9 T7 parental (+/+), knockout (-/-) and addback (-/-/+) cell lines. (a, b) *ΔPF16*, (c, d) *ΔIFT88* and (e, f) *ΔBBS2*. (a, c, e) Measurement of footpad size over time of infection. The average from five mice is shown with error bars indicating standard deviation. *p*-values are indicated for the 8 weeks post-infection time point and were calculated using a two-tailed unpaired (unequal variance) Students’ t test. (b, d, f) Parasite burden measured from serial dilutions from footpad lesions and lymph nodes. The average is indicated. *p*-values were calculated using a Kruskal-Wallis-test with Dunn’s post-hoc test and Bonferroni correction, comparing knockout and addback against each other (asterisks on line) and against the parental (individual asterisks). (*p*-value: *<0.05, **<0.0001; ns: non-significant; raw values and *p*-values are provided in the Supplementary Table S4).

The footpads of mice infected with the parental cell line showed a progressive increase of lesion size. Lesion sizes from mice infected with the Δ*PF16* mutant initially progressed in a similar way to the parental cell line and to the *PF16*-AB cell line, which had restored PF16 expression (Supplementary Figure S1g). At the 8-week end point, there was a small difference in lesion size between mice infected with the parental cell line and the Δ*PF16* mutant. Infections with the addback cell-line *PF16-AB* restored lesion sizes closer to the values obtained with the parental line (Figure 2a). The parasite burden in the footpad and lymph node was calculated from serial dilutions of extracted tissue. The presence of growing parasites from all samples was noted after 3 weeks in culture, indicating that live parasites were recovered from mice infected with the Δ*PF16* mutant and the *PF16-AB* cell line from the footpad lesions and from the lymph nodes (Figure 2b). The calculated parasite burden was, however, lower for the Δ*PF16* mutants, compared to the parental line. These results show that despite being paralysed, the PF16 knockout parasites were able to establish footpad lesions, persist for 8 weeks in mice and remain viable and competent for differentiation to promastigote forms.

### IFT88 mutants, which lack external flagella, fail to establish an infection

In mice infected with Δ*IFT88* parasites, no lesion growth was seen, while the mice infected with the parental cell line (Cas9 T7) showed a progressive increase in lesion size (Figure 2c). By contrast, the *IFT88*-AB cell line appeared to have the same pathogenicity as the parental cell line, with similar footpad sizes (Figure 2c). Eight weeks after the challenge, the mice were culled and dissected, and serial dilutions were performed to estimate parasite burden in footpads and lymph nodes after 3 weeks of culture. Growing parasites were noted for the parental and the IFT88-AB cell line but no parasites were recovered from the footpad lesion and lymph nodes from mice infected with the Δ*IFT88* cell line (Figure 2d). These data show that *IFT88* null mutants are avirulent; they are incapable of causing footpad swelling and likely perish in the mouse. Re-introduction of the *IFT88* gene, which restores flagellar growth [12], was sufficient to restore pathogenicity.

### The BBSome protein BBS2 is essential for amastigotes

The Δ*BBS2* mutant also failed to produce footpad swelling, in stark contrast to the expected increase of lesion size in mice infected with the parental cell line (Figure 2e). Lesions were noted after infections with the *BBS2-AB* cell line and the footpads grew in size similarly to the parental control infections (Figure 2e). Following serial dilutions of dissected footpad and lymph node tissue after 8 weeks of infection, growing parental and *BBS2-AB* parasites were noted after 3 weeks in culture, and the number of parasites per lesion and lymph node was calculated (Figure 2f). No Δ*BBS2* mutants were recovered from the footpad lesions nor from the lymph nodes (Figure 2f). These data show that *BBS2* is required for parasite pathogenicity.

The detailed observations on mice infected with individual cell lines support conclusions from the pooled screen, namely that *BBS2* and *IFT88* null mutants are significantly attenuated in their pathogenicity compared to the parental cell line. Virulence was restored by re-introducing the respective genes into the knockout parasites. Footpad measurements and parasite burden calculations for both cell lines indicated they lack the ability to infect the mammalian host. By contrast, the *PF16* null mutant was able to infect the mouse and also had the ability to back transform from amastigotes to proliferative promastigotes.

## Discussion

Here, we assessed the pathogenicity of *L. mexicana* lines that have been genetically engineered to have distinct deficiencies in flagellum structure or function. The pooled bar-seq screen indicated that perturbations of motility through targeted removal of axonemal proteins (listed in Supplementary Table S1) had little effect on parasite fitness *in vivo*. Parasites exhibiting a range of motility defects that varied in severity from altered swim speed (increased or decreased, Figure 1a) to complete paralysis remained detectable in infected macrophages for 8 d and in footpad lesions for 6 weeks, with no apparent change in abundance compared to the parental control cell lines (Figure 1d,e). The *PF16* knockout cell line was chosen to verify this finding on the basis of an individual mutant, since its phenotype is already well-characterized in promastigote forms *in vitro* and in the sand fly [12,22]. Here, despite its inability to displace itself or produce any sustained flagellar beats, the Δ*PF16* mutant was able to establish footpad infections on its own, clearly demonstrating that a beating flagellum is not required for infection, nor for subsequent survival in an animal host. Between ~6 and 8 weeks after infection, footpad lesions appeared to plateau in the mice infected with the Δ*PF16* mutant, while still increasing in the control infections. This is consistent with the lower calculated parasite burden at the final time point. Whether this indicates enhanced parasite clearance or a reduced parasite proliferation rate *in vivo* is unclear and merits further investigation. The fact that viable *PF16* knockout parasites were recovered after 8 weeks of infection and that these were still capable of transforming back to promastigote forms indicates that the stalling of lesion development is not due to effective parasite clearance.

Taken together, the phenotypes of five different mutants with severe motility defects arising from the loss of core axonemal proteins (Δ*CFAP43*, Δ*CFAP44*, Δ*RSP4/6*, Δ*IC140* and ΔPF16) which all showed normal fitness in the pooled infection assays, and which was confirmed by the individual infections with Δ*PF16*, shows that motility is dispensable for pathogenicity. While it is known that some motility defects can be overcome by secondary mutations [43], it seems unlikely that such mutations would have arisen spontaneously with sufficient frequency in all of these different mutants to account for the observed results. Moreover, the finding that motility is dispensable for *Leishmania* in the mammalian host fits well with the naturally occurring remodeling of the axoneme during amastigote differentiation, which produces non-motile cells: the amastigote 9 v axoneme lacks the paraflagellar rod and axonemal dynein motors required for generating a flagellar beat [37,39,44]. In this respect, *Leishmania* differs from other kinetoplastid pathogens. In *Trypanosoma cruzi*, the intracellular amastigotes retain a 9 + 2 axoneme flagellum shown to beat with ~0.7 hz inside host cells [45]. Its function remains a matter of speculation [46] and it would be interesting to test the consequences of interfering with this movement to discover if it is required for pathogenicity. The flagellum of *T. brucei* bloodstream forms is motile, and several studies have demonstrated that perturbations of the flagellum lead to rapid cell death *in vitro* [8,47] and promote parasite clearance from the mammalian host [18]. These findings may aid the search for new drugs against African trypanosomiasis [2]. For *Leishmania*, an essential requirement for flagellar motility has only been shown for promastigotes in their sand fly vector [12].

Interestingly, however, the results with the Δ*IFT88* mutant reported here indicate that assembly of a flagellum, even if paralysed, or extending only a short distance out of the flagellar pocket, is necessary for successful infection of a mammalian host. IFT88 is a conserved component of the IFT-B complex, which is required for the assembly of microtubule axonemes in most ciliated species. *L. mexicana* promastigotes that lack IFT88 or other IFT proteins can grow in culture, albeit with a slightly longer doubling time of ~10 h compared to the ~7 h in the wild type [12,21]. They were, however, unable to establish a sand fly infection [12] and the results from the present study show they are unable to infect mice.

Why are the IFT mutants avirulent? A structural role for the flagellum in flagellar pocket architecture could explain this. In promastigotes, the flagellar pocket membrane is attached to the flagellum membrane on one side, leaving a relatively wide opening allowing for fluid access. By contrast, the amastigote flagellar pocket neck closely adjoins the flagellar membrane on all sides, reducing the width of the opening by nearly 10-fold compared to the promastigote FP [48]. Disruption of the flagellar pocket architecture by deletion of flagellar attachment zone protein 5 (FAZ5) has been shown to attenuate pathogenicity in experimental mouse footpad infections [49]. While the flagellum is not essential for the formation of the flagellar pocket itself [21], it could serve to protect the flagellar pocket lumen from harmful host factors and contents of the parasitophorous vacuole. The Δ*FAZ5* phenotype would support this hypothesis, since these mutants had a wider FP opening and were slightly more sensitive to the lethal effects of whole mouse serum compared to the parental cell line [49]. The Δ*IFT88* mutant may be similarly sensitive. The morphological consequences of the IFT disruption were studied in *L. mexicana* promastigotes following deletion of the IFT dynein heavy chain (DHC2.1), which powers retrograde transport [50] and IFT-A complex protein IFT140 [21]. These analyses revealed that the mutants still formed a flagellar pocket, which surrounded a short stump of a cilium with a short collapsed 9 + 0 axoneme that terminated before reaching the flagellar pocket collar. While this raised the interesting question of whether the formation of the 9 v amastigote cilium is indeed IFT-dependent or instead triggered by the absence of IFT [21], the amastigote flagellum is structurally more complex than the rudimentary 9 + 0 axoneme in IFT deletion mutants. It terminates a short distance beyond the flagellar pocket collar in a bulbous flagellum tip that contains electron dense material of unknown composition [39,48]. In a naturally occurring *L. braziliensis* strain with an unusually short flagellum, a normal structural architecture of the flagellar neck region was maintained and this strain remained infection-competent [51].

An alternative explanation for how the amastigote flagellum might benefit the parasite is by serving as a point for signal transduction between the parasite and host cell. In mice, IFT and BBS2 deletion mutants exhibited a very similar loss of pathogenicity both in the pooled bar-seq screen (Figure 1d) and in the individual infection experiments (Figure 2). This is compatible with a model where the IFT and BBSome functions are linked, as is the case in other cilia where the BBSome acts as a cargo adaptor for IFT, specifically promoting the removal of ciliary proteins [52]. BBSome proteins are highly conserved in ciliated eukaryotes [10,53,54]. Losing BBSome function was shown to impact on the localization of ciliary membrane proteins in phylogenetically diverse species [55–59], with detrimental consequences for ciliary signal transduction. Biochemical studies in *T. brucei* confirmed the direct interaction of BBS proteins in a ~ 700 kDa complex [60], and BBS proteins were located at the flagellar transition zone, the gate that controls entry to the ciliary compartment [61]. In *Lottima passim*, a kinetoplastid parasite of honey bees, the flagellar calcium-binding protein FCaBP was found by biotin-proximity labeling to be located close to BBS1 [62]. While the IFT-like movement of BBS proteins along the flagellum has not been observed in trypanosomes, this absence of evidence could reflect the technical challenge of detecting low abundance proteins by live cell microscopy [61]. Flagellar length was reduced upon depletion of Arl6 (BBS3) by RNAi in *T. brucei* [63], but flagellum assembly and motility remained normal in the study by [60] and motility was normal in *L. mexicana* BBSome deletion mutants (Figure 1a). Changes in the *T. brucei* cell surface composition were, however, noted and the mutants took up less transferrin compared to the wild type [60]. Overall, this evidence is compatible with a model where the BBSome in kinetoplastids acts at the flagellum base to sort-specific membrane proteins [6,60], in line with the established function of the BBSome in a range of organisms. What the identity, origin and destination of the sorted proteins is, and whether any have specific functions in the ciliary membrane, is currently not clear. At what point during the differentiation from promastigote to amastigotes the parasites become highly sensitive to the loss of the BBSome cannot be definitively concluded from the data. Perturbations of membrane protein trafficking are likely to have multiple detrimental effects, including delivery of amastigote-specific molecules to the cell surface. We can speculate that this would lead to impaired signal transduction, misregulation of solute and nutrient transport and perhaps reduced secretion of virulence factors. What is clear is that processes governed by the BBSome are important for the pathogenicity of several kinetoplastid parasite species. The importance of BBSome proteins for the virulence of two other kinetoplastid species has previously been shown, in *T. brucei* [60] and *L. major* (LmjBBS1 [64]). Here, a different core subunit of the BBSome, targeted in a different *Leishmania* species, resulted in an equally severe loss-of-fitness phenotype. This severe phenotype was seen in experiments where the BBS2 mutant was present alone (Figure 2) as well as in the pooled infections (Figure 1), indicating that the presence of BBS2 wild-type cells did not protect the BBS2 knockout cells. Our results thus replicate and extend the interesting findings by Price *et al*. consolidating the evidence for an indispensable function for the BBSome in *Leishmania* pathogenicity.

## Materials and methods

### Cell culture

Promastigote-form *L. mexicana* Cas9 T7, which were generated in the laboratory from WHO strain MNYC/BZ/62/M379 [22], were grown at 28°C in M199 medium (Life Technologies) supplemented with 2.2 g/L NaHCO_3_, 0.005% hemin, 40 mm 4-(2-Hydroxyethyl)piperazine-1-ethanesulfonic acid (HEPES) pH 7.4 and 10% FCS.

### Genetic modification of *L. mexicana*

CRISPR-Cas9 gene knockouts were made as described in Beneke T, et al. (2017) [22]. Briefly, the oligo sequences from www.LeishGEdit.net were used for the design of sgRNA templates and donor DNA cassettes. The *L. mexicana* Cas9 T7 parental cell line containing the *S. pyogenes* Cas9 gene encoded on pRM006 and T7 RNA Polymerase on pVY087 [22,65] was transfected in a single-step transfection with sgRNA templates and donor DNA cassettes derived from pTNeo and pTPuro (BBS2; PF16) or pTNeo and pTBlast (IFT88) and selected with 40 µg/ml G418, 20 µg/ml Puromycin or 5 µg/ml Blasticidin, as appropriate, in supplemented M199 medium. For the addback cell lines, the coding sequences (CDS) of PF16 (LmxM.20.1400) and IFT88 (LmxM.27.1230) were cloned in plasmid pTAdd (which was derived from pRM005 [22] by replacing the Cas9 gene with restriction enzyme recognition sites to facilitate insertion of coding sequences). The coding sequence of BBS2 was found to be incorrectly annotated on TritrypDB, starting from a CDS-internal methionine. The full-length BBS2 coding sequence was identified by aligning the protein sequences of the conserved BBS2 sequences from *H. sapiens* and *T. brucei* to the translated in-frame ORF upstream of the annotated start codon of LmxM.29.0590. This identified a methionine corresponding to the start codon in the other proteins. This 176 amino acid extension was compatible with the mapped splice acceptor and poly-A sites from *L. mexicana* RNA-seq data [66], and this longer BBS2 CDS (Supplemental Data File) was used to generate the addback plasmid. The sequences of all inserted CDS were checked by Sanger sequencing. The null mutant cell lines were transfected with the relevant addback plasmids and selected with 25 µg/ml Phleomycin in supplemented M199 medium at 28ºC.

### Diagnostic PCR for knockout validation

Genomic DNA was isolated as previously described by Rotureau B. et al. [67]. Primers for diagnostic PCRs were designed using Primer3 [68,69]. Diagnostic PCRs to test for the presence of the target ORF in the putative KO lines and the parental cell lines used in the pooled assays were reported in [12]. All cell lines used in the current study were again tested by PCR before pooling them for the *in vivo* experiments. Since the SBL1-5 cell lines were generated by replacing the nourseothricin-resistance gene within the 18S rRNA SSU locus with a barcoding cassette [12], the absence of the resistance gene was confirmed as part of the diagnostic PCR. The cell lines used for individual *in vivo* infections were also tested for the deletion of the *PF16*, *BBS2* and *IFT88* ORF, respectively, as well as for the presence of the reintroduced ORF in each addback cell line. All used primers are listed in Supplementary Table S3. To show the presence of DNA, a second PCR reaction was performed to amplify the ORF of blasticidin-S deaminase. SBL barcoded parental cell lines were verified by amplifying the ORF of streptothricin acetyltransferase.

### Pooling of mutants for bar-seq screens

For mouse and BMDM infections, sub-pools were seeded at different starting densities, according to their growth rates as promastigotes [12], to ensure that each mutant in the pool would be equally represented in the stationary phase cultures used for the infections. Sub-pools were seeded at 6 × 10^6^ cells/ml (pool 1; ΔPMM, ΔGDP), 3 × 10^6^ cells/ml (pool 2; *ΔIFT88*, *ΔARL-3A*, *ΔLPG1*, *ΔPMI*), 1.5 × 10^6^ cells/ml (pool 3; *ΔIC140*, *ΔPF16*, *ΔCFAP44*, *ΔCFAP43*, *ΔRSP4/6*, *ΔdDC2*, *ΔLmxM.21.1110*, *ΔLC4-like*, *ΔFM458*, *ΔPDEA*, *ΔLmxM.23.1020*, *ΔLmxM.17.0870*, *ΔBBS2*, *ΔKHARON1*) or 1 × 10^6^ cells/ml (pool 4; SBL1–5), respectively, and grown for 96 hours. Just before mouse or BMDM infection, the sub-pools were then mixed in proportions to ensure equal representation of mutant lines, and a DNA sample was extracted from 1 × 10^7^ cells with the Qiagen DNeasy Blood and Tissue Kit.

### Bone marrow-derived macrophage (BMDM) differentiation and infection

Murine bone marrow cells were harvested from the femurs and tibias of BALB/c mice. Bone marrow cells were allowed to differentiate *in vitro* in DMEM supplemented with 20% L929, 10% FBS (Gibco) and 1% Penicillin/Streptomycin (Sigma) for 7 d at 37°C, 5% CO_2_. Flow-cytometry was used to confirm that the cells were positive for the murine macrophage markers F4/80 (Alexa Fluor488-conjugated antibody, clone BM8) and MAC-1 (Alexa Fluor488-conjugated antibody, clone M1/70), and negative for the granulocyte marker GR-1 (Alexa Fluor647-conjugated antibody, clone RB6-8C5). BMDMs were cryopreserved until use in 90% FBS plus 10% DMSO. In preparation for infection, BMDMs were thawed, seeded in 24-well plates (2 × 10^5^ cells per well) and incubated in 1 ml DMEM differentiation medium for 6 h at 37°C, 5% CO_2_. The medium was then replaced with fresh pre-warmed DMEM supplemented with 10% FBS and 1% Penicillin/Streptomycin and the plates were incubated at 34°C, 5% CO_2_ for 24 h before adding the *Leishmania*. The pooled stationary phase *Leishmania* cell lines were counted, and the parasite density was adjusted to 1 × 10^6^ parasites/ml in supplemented DMEM resulting in a 1:5 ratio of parasites to macrophages. After a 3 h incubation at 34°C, the parasites were washed from the macrophages, and freshly supplemented DMEM was added. Time points 3 h, 24 h, 48 h, 96 h and 192 h were taken, and DNA was extracted using the Qiagen DNeasy Blood and Tissue Kit.

### Mouse infections and DNA extraction

Animal work was carried out according to the Animals (Scientific Procedures) Act of 1986, United Kingdom, and was approved by the University of York Animal Welfare and Ethical Review Body (AWERB) committee. BALB/c mice were purchased from Charles River Laboratories and maintained in a pathogen-free facility at the University of York. All mice used in the experiments were socially housed under a 12 h light/dark cycle and were only used in this experiment. For each time point, six female 6-week-old BALB/c mice were subcutaneously infected in the left footpad with stationary promastigote pools using either 2 × 10^6^ parasites (high dose) or 2 × 10^5^ parasites (low dose) in 40 μl of sterile PBS. The researchers handling the animals were blind to the phenotype of the mutant *Leishmania*. Footpad swelling was measured [70] and increased generally from 1.8 mm to 2.5 mm over 6 weeks. Three and 6-week post-challenge, the mice were euthanised and infected footpad and popliteal lymph nodes were dissected. Tissues were stored at −20°C until the DNA was extracted using the DNeasy Blood and Tissue DNA kit (Qiagen). Samples were incubated for 2 h at 56ºC in the ATL tissue lysis buffer provided with the kit and Proteinase K (Qiagen) to allow complete lysis, then the manufacturer’s instructions were followed.

### Illumina library preparation and sequencing

Sequencing libraries were prepared as described in Beneke, et al. [12]. Briefly, for each sample, 600 ng of isolated DNA was treated with exonuclease VII (NEB) and purified using SPRI magnetic beads. Barcode regions were amplified with custom-designed p5 and p7 primers (Life Technologies), containing indexes for multiplexing and adapters for Illumina sequencing. Indices were derived from Illumina Nextera (indices 501–517) and TruSeq (indices RPI1-RPI48) indexing kits [71]. Bead-purified amplicons were pooled in equal proportions, and the pool was diluted to 4 nM and spiked with 30% single indexed *Leishmania* genomic DNA and 1% PhiX DNA; 8 pM was sequenced using a MiSeq v3 150 cycle kit following the manufacture’s instructions with paired-end sequencing (2 × 75 cycles, 6 and 8 cycle index read).

### Bar-seq data analysis

Data analysis followed the process described in Beneke et al. [12]. MiSeq raw files were de-multiplexed using bcl2fastq (Illumina). The occurrence of each barcode in the sequencing reads was counted using a bash script [71], searching against the whole database of LeishGEdit barcodes and counting only barcodes with a 100% match to the 17 nt total length. Counts for each barcode were normalized for each sample by calculating their abundance relative to the total number of reads for all 25 barcodes included in the pool. To calculate “fitness”, normalized barcode counts in the pooled population before infection were divided by normalized counts at the relevant time point post-infection.

### Infection of mice with individual *L. mexicana* lines and quantification of parasite burden

All cell lines (Δ*BBS2, BBS2-AB*, Δ*PF16*, *PF16-AB*, Δ*IFT88* and *IFT88-AB)* were confirmed to be knockouts via PCR diagnostics. Once confirmed to be null mutants and re-expressors, each cell line, including the parental *L. mexicana* Cas9 T7, was grown to stationary phase promastigotes, left for 3 d and then used to inoculate the footpad of BALB/c mice at a density of 2.5 × 10^6^ per mouse. Individual cell lines were anonymously inoculated (five mice per cell line), and the order of measurements was not controlled. Virulence of the cell lines was monitored via weekly footpad measurements. The footpad lesion of the control cell lines was allowed to progress to approximately 3 mm. Eight weeks after the challenge, the mice were euthanized, infected footpads and popliteal lymph nodes were dissected and macerated. The tissues were mechanically dissociated and filtered through a 70 μm cell strainer. Homogenates were resuspended in HOMEM supplemented with 20% FBS (Gibco) and 1% Penicillin/Streptomycin (Sigma), and serial dilutions were performed. These plates were incubated at 25°C for approximately 3 weeks. The presence of growing parasites was noted and parasite burden was calculated. No animals or data points were excluded from the study. The footpad measurement data passed the Kolmogorov-Smirnov test of normality and *p*-values were calculated using a two-tailed unpaired (unequal variance) Students’ t test. The parasite burden data were analysed with a Kruskal-Wallis test with Dunn’s post-hoc test and Bonferroni correction. Sample size calculations were done with the significance level set at 5% power at 80% and a two-sided test selected. Under the assumption that mutants with changes in virulence result in a 50% difference in the footpad lesion size at 8 weeks after infection, a minimum of four animals was required per group for statistical power.

## Supplementary Material

QVIR-2025-0130.R1- clean copy of supplementary table 4.xlsx

Beneke_Neish_et_al_Supplementary_Data_Figure_S1.tif

Beneke_Neish_et_al_Supplementary_Table_3.xlsx

Beneke_Neish_et_al_Supplementary_Table_1.xlsx

Beneke_Neish_et_al_Supplementary_Table_2.xlsx

## Data Availability

The authors have adhered to ARRIVE guidelines and confirm that all the data supporting the findings of this study are available within the article and its supplementary materials, which have been deposited to Figshare (https://figshare.com/) under https://doi.org/10.6084/m9.figshare.28601786.v1 (Supplementary Figure S1) https://doi.org/10.6084/m9.figshare.28601825.v1 (Supplementary Tables 1–4) https://doi.org/10.6084/m9.figshare.28601846.v1 (Supplementary Data File).
