# Supplementary figures and images for: Leishmania mexicana pathogenicity requires flagellar assembly but not motility
Source: Virulence. 2025 Jul 2;16(1):2521478. doi: 10.1080/21505594.2025.2521478 (PMC12233823; doi:10.1080/21505594.2025.2521478)

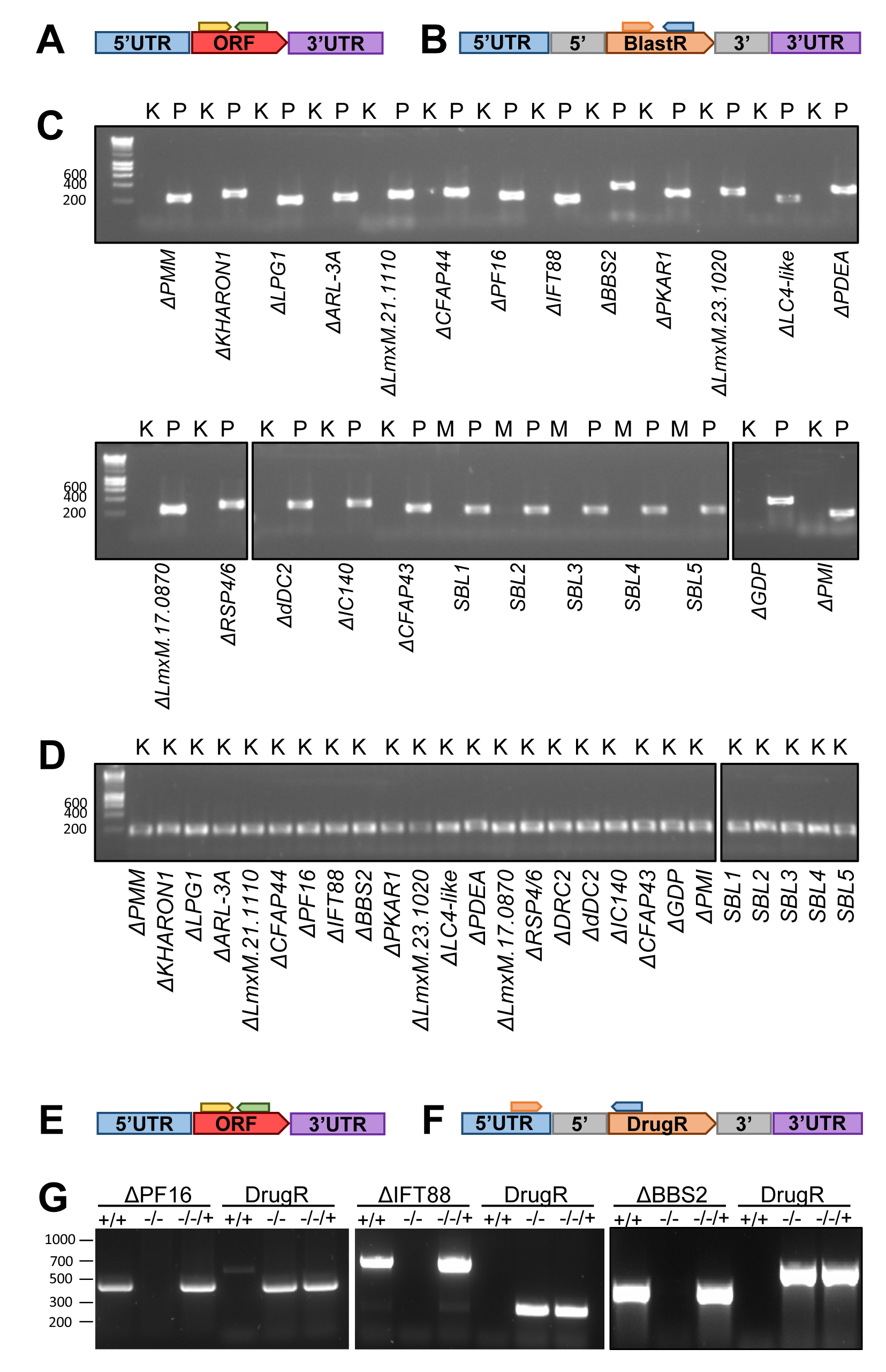

Supplement: Beneke_Neish_et_al_Supplementary_Data_Figure_S1.tif [file KVIR_A_2521478_SM2773.tif]
